# Employing intrinsic fluorone-dye fluorescence in a quenching-based (“on-off”) strategy for diltiazem determination in tablets and capsules

**DOI:** 10.1186/s13065-025-01654-4

**Published:** 2025-12-01

**Authors:** Ahmed A. Abu-hassan

**Affiliations:** Department of Pharmaceutical Analytical Chemistry, Faculty of Pharmacy, Al-Azhar University, Assiut Branch, Assiut, 71524 Egypt

**Keywords:** Diltiazem hydrochloride, Spectrofluorimetric, Validation, Green method, Pharmaceutical formulations

## Abstract

A novel green spectrofluorometric method was developed for the quantification of diltiazem hydrochloride (DLZ), a benzothiazepine-class calcium channel blocker with vasodilatory properties. The assay exploits the rapid fluorescence quenching of Acid Red 87—a fluorone-based dye—upon complexation with DLZ in acidic medium (pH 3.8). This “on-off” mechanism enables selective DLZ detection by measuring the decrease in Acid Red 87 native fluorescence intensity (λ_ex_/λ_em_ = 302.5/545.8 nm). Key parameters (pH, dye concentration, buffer volume) were systematically optimized, yielding a linear response over 50–1100 ng/mL (r² = 0.9991) with a detection limit of 15.5 ng/mL. The method was rigorously validated per ICH Q2(R1) guidelines, confirming precision (RSD < 2%), accuracy (99.76% % recovery), and robustness. It was successfully applied to analyze DLZ in pharmaceutical formulations (tablets/capsules) with no matrix interference, and the statistical comparison (t- and F-tests) showed no significant difference from the reference method. Critically, the procedure uses distilled water as the sole solvent, aligning with green chemistry principles while offering simplicity, cost-efficiency, and high-throughput potential.

## Introduction

 Diltiazem HCl (DLZ), a benzothiazepine-class calcium channel blocker (CCB), mediates its therapeutic effects primarily by inhibiting voltage-dependent calcium influx in cardiac and vascular smooth muscle cells during depolarization. This suppression of calcium translocation modulates myocardial contractility and vascular tone, underpinning its antihypertensive, antianginal, and antiarrhythmic efficacy [1, 2]. Unlike dihydropyridines (e.g., nifedipine) with pronounced vascular selectivity or phenylalkylamines (e.g., verapamil) with marked cardiac activity, DLZ exhibits balanced cardio-selectivity, influencing both tissue types. Consequently, DLZ is clinically indispensable for managing hypertension, chronic stable angina, atrial fibrillation, and atrial flutter. Off-label applications further extend to pulmonary hypertension, migraine prophylaxis, anal fissures, and nocturnal leg cramps, reflecting its broad pharmacologic utility [3–6].

Given its extensive clinical use, rigorous quality control of DLZ in pharmaceutical formulations and therapeutic monitoring in biological matrices is imperative. Analytical methodologies for DLZ quantification—spanning chromatographic (HPLC, LC-MS) [7–9], HPTLC [10], electrochemical (voltammetry) [11, 12], and spectrophotometric (UV/Vis) [13–15] techniques—have been comprehensively reviewed. While spectrofluorometric approaches exploiting luminescent reactions exist, a significant gap remains: only one validated fluorometric method currently enables DLZ determination in pharmaceutical samples with adequate sensitivity and environmental sustainability.

Spectrofluorometry is a well-established analytical platform for drug quantification in both pharmaceutical formulations and biological matrices, valued for its exceptional sensitivity and operational simplicity. The present fluorimetric strategy offers distinct advantages over chromatographic separations (e.g., HPLC), notably through its instrumental economy, minimal procedural requirements, and elimination of costly organic solvent consumption. Unlike HPLC—which demands significant technical expertise and generates substantial hazardous waste—this approach utilizes affordable reagents and circumvents complex extraction protocols or thermal derivatization. Furthermore, spectrofluorimetry demonstrates superior analytical performance compared to conventional UV-Vis spectrophotometry, achieving enhanced selectivity and lower detection limits without compromising accessibility or throughput [16–19].

Xanthene dyes represent a valuable class of fluorophores in analytical science due to their high quantum yields, biomolecular affinity, and versatile applications. Acid Red 87, a halogen-substituted fluorone derivative, exemplifies these attributes through its utility as a laser dye, histological stain, and diagnostic probe. Its intrinsic photoluminescence and selective biomolecular interactions underpin established quantitative methods for proteins in biological matrices. Furthermore, Acid Red 87 serves as an effective fluorescence probe for pharmaceuticals, demonstrated in assays for asenapine [16], vonoprazan [20], eletriptan [21], β-blocker [22], and fingolimod [23]. This precedented utility motivated its adoption as a fluorogenic reporter for diltiazem HCl (DLZ) quantification in the present study. The developed method underwent comprehensive validation per ICH Q2(R1) guidelines, confirming reliability for DLZ determination in pharmaceutical tablets and capsules. Critically, the assay exemplifies green analytical chemistry principles: distilled water serves as the exclusive solvent, eliminating organic waste while maintaining high sensitivity.

## Experimental

### Apparatus

Fluorescence measurements were acquired using an FS-2 spectrofluorometer (SCINCO Co., Korea) equipped with a 150 W xenon arc lamp. The slit width was adjusted to 10 nm. Solution pH was monitored using an AD11P digital pH meter (Adwa Instruments, Romania), calibrated daily with standard buffers. All weightings employed a calibrated analytical balance (Mettler Toledo XPR205DR, resolution 0.01 mg) operating within controlled ambient conditions.

### Materials and reagents

Diltiazem hydrochloride (DLZ; certified purity 99.8%) was provided by EIPICO (Tenth of Ramadan City, Egypt). Acid Red 87; fluorone dye was sourced from Sigma-Aldrich (UK). Pharmaceutical formulations—Altiazem^®^ 60 mg tablets and Diltiazem 90 mg diltiazem HCl capsules—were procured from commercial pharmacies (Tanta, Egypt). Citric acid, phosphoric acid, and sodium hydroxide (analytical grade) were obtained from El-Nasr Pharmaceutical Chemicals Co. (Cairo, Egypt). HPLC-grade methanol was purchased from Fisher Scientific. All solutions were prepared in ultrapure water.

### Stocks solutions

A DLZ stock solution (200 µg/mL) was prepared by dissolving 20.0 mg diltiazem hydrochloride (EIPICO, 99.8% purity) in ultrapure water within a 100-mL Class A volumetric flask, followed by dilution to the mark. Working standards (0.5–11 µg/mL) were obtained by serial dilution of the stock solution in water. All solutions were stored at 4 ± 1 °C in amber glass vials and used within 24 h to ensure stability.

### Teorell-Stenhagen buffer preparation

Universal buffer solutions (pH 2.0–12.0) were formulated using a modified Teorell-Stenhagen protocol:

- Solution A: contains specified volumes of 0.33 M citric acid, 0.51 M phosphoric acid, and 1 M NaOH.
- Solution B: 0.10 M HCl.
- Target pH values were achieved by mixing Solutions A and B in predetermined ratios, verified using a calibrated pH meter (Adwa AD11P).

### Optimization of experimental conditions

To achieve optimal performance with the fluorimetric method, it was essential to systematically assess and refine a range of experimental parameters expected to affect the fluorescence intensity readings. Key aspects such as the choice of excitation and emission wavelengths, the configuration of the instrumentation, and critical system conditions—including pH levels and the volumes of buffer and fluorescent dye—were rigorously investigated. Each variable was carefully optimized to improve the reliability and precision of the ∆FI data. These deliberate adjustments contributed to the overall robustness of the fluorimetric technique, ensuring its capacity to yield consistent and meaningful analytical results.

### Impact of pH and buffer amount

**Fig. 1:**
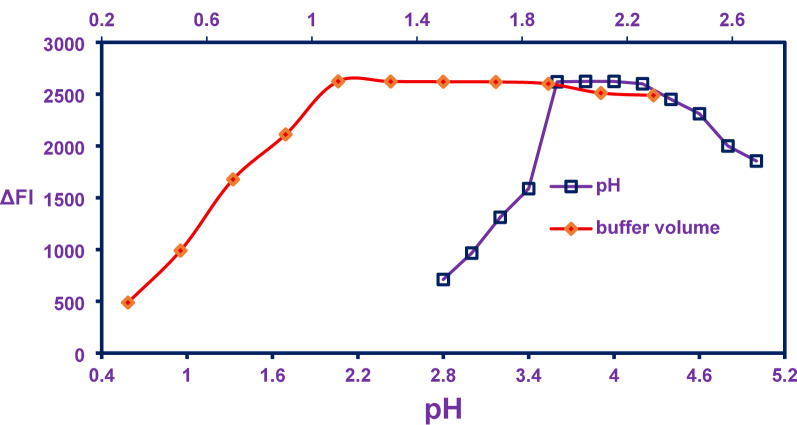
Impact of different pH and buffer volumes in the outcomes of RFI

To establish the proposed spectral approach, the impact of pH on the interaction between DLZ and Acid Red 87 was systematically explored within the pH range of 2.8 to 5.0. The results demonstrated a pronounced dependence of complex formation on pH, with the most substantial changes in fluorescence intensity (ΔFI) observed between pH 3.6 and 4.0. Based on these findings, a pH of 3.8 was identified as the optimal condition for subsequent experiments, as illustrated in Fig. 1.

Further assessment of the fluorimetric method’s reliability involved evaluating the effect of varying volumes of Torell buffer—ranging from 0.3 to 2.3 mL—on the binding efficiency between DLZ and Acid Red 87. The data revealed that buffer volume significantly influenced complex formation, with maximal ΔFI values recorded when buffer volumes were maintained between 1.1 and 1.7 mL. Excessive buffer volumes appeared to introduce competitive interactions between the buffer’s positively charged constituents and DLZ cations, thereby impeding complexation. Conversely, insufficient buffer volumes failed to adequately stabilize the pH, compromising the reaction conditions. As a result, a buffer volume of 1.3 mL was selected as the optimal parameter for the experimental setup, as shown in Fig. 1.

### Execution of analytical procedures

Aliquots (1.0 mL) of DLZ working standards (0.5–11 µg/mL) were transferred to 10-mL volumetric flasks. Each flask received 1.2 mL of Acid Red 87 (0.03 w/v in H₂O) and 1.3 mL of Teorell-Stenhagen buffer (pH 3.8 ± 0.1). Solutions were vortex-mixed (10 s), diluted to volume with ultrapure water, and equilibrated for 5.0 ± 0.5 min at 25 °C. A reagent blank (Acid Red 87 + buffer, no DLZ) was prepared concurrently. Fluorescence quenching value was monitored by measuring the decrease in Acid Red 87 native fluorescence intensity (λ_ex_/λ_em_ = 302.5/545.8 nm) using a SCINCO FS-2 spectrofluorometer (slit widths: 10 nm).

### Dosage forms analysis

Ten tablets of Altiazem^®^ (60 mg DLZ/tablet) or the contents of ten capsules (90 mg DLZ/capsule) were accurately weighed and finely triturated. A powder aliquot equivalent to 20.0 mg diltiazem HCl was transferred to a 100-mL volumetric flask and dissolved in 50 mL ultrapure water via sonication (10 min, 25 °C). The solution was filtered through a 0.45-µm nylon membrane, diluted to volume with water, and centrifuged (4000 rpm, 10 min). The supernatant was diluted quantitatively to yield concentrations within the working range (0.5–11 µg/mL). DLZ content was determined spectrofluorometrically using the established calibration curve (y = 4.42x + 1148.5).

### Procedure for estimating the molar ratio of the complex

An investigation into the binding stoichiometry between Acid Red 87 and DLZ was conducted via Job’s method of continuous variation [24]. Separate master solutions of DLZ and Acid Red 87 were prepared at identical concentrations of 2.5 × 10⁻⁴ M. A Series of samples was created by mixing these solutions in various complementary volume ratios. Following the application of the proposed procedure, the measured ΔRFI for each sample was adjusted by subtracting a blank reading (in the absence of drug). The resulting corrected ΔRFI was then graphed against the corresponding mole fraction of DLZ to determine the complex ratio.

## Results and discussion

**Fig. 2:**
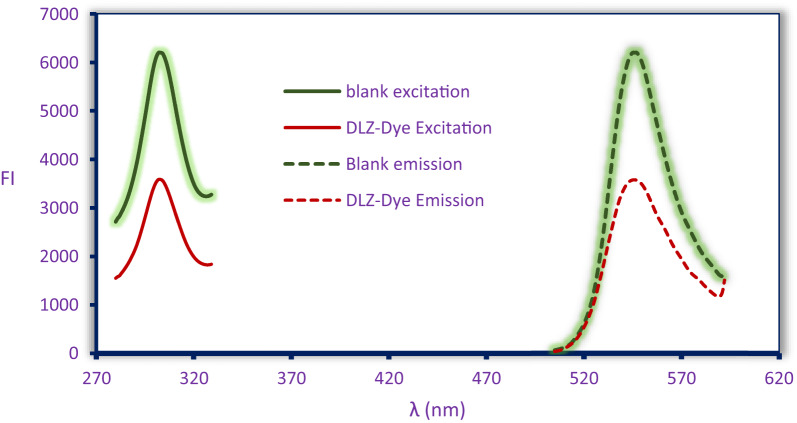
The scanned spectrum of DLZ-Acid Red 87 complex and blank

**Fig. 3:**
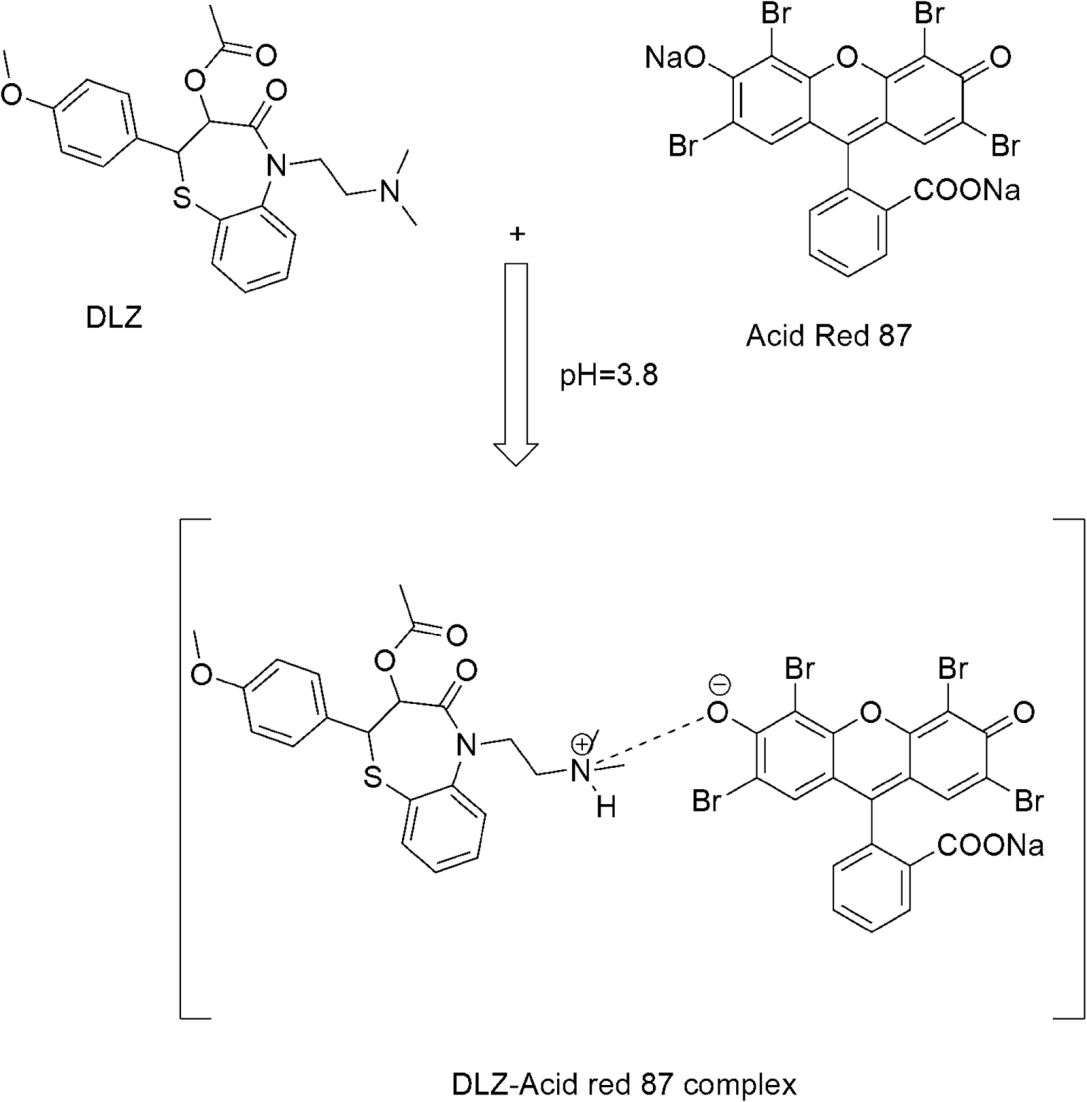
The structural pathway of DLZ- Acid Red 87 complex formation

Xanthene dyes are valued in analytical chemistry for their high quantum yields, biomolecular affinity, and versatile applications. It is demonstrated in assays of asenapine [16], vonoprazan [20], eletriptan [21], β-blocker [22], and fingolimod [23]. This work employs Acid Red 87, a halogenated fluorone dye, as a fluorogenic probe for diltiazem HCl (DLZ) quantification. Acid Red 87 exhibits native fluorescence (λ_ex_/λ_em_ = 302.5/545.8 nm) that undergoes concentration-dependent quenching upon DLZ interaction (Fig. 2). The underlying mechanism involves ion-pair complex formation between protonated DLZ (at pH 3.8) and the anionic Acid Red 87 species, driven by electrostatic attraction (Fig. 3). Complex stability is governed by charge density, intermolecular distance, and solvation effects, which collectively modulate fluorescence extinction. This phenomenon enables selective DLZ quantification across 50–1100 ng/mL.

The method was validated by ICH Q2(R1) guidelines and successfully applied to DLZ determination in pharmaceutical tablets (Altiazem^®^ 60 mg; Diltiazem 90 mg capsules) with content uniformity verification. Crucially, the assay adheres to green chemistry principles by using distilled water as the exclusive solvent, eliminating organic waste while maintaining analytical robustness.

### Acid red 87 dye volume

**Fig. 4:**
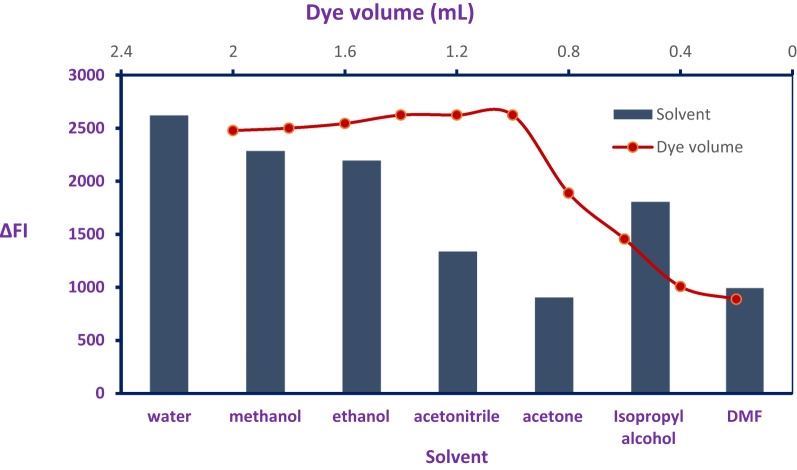
Impact of different volumes of Acid Red 87 and dispersing solvents in the outcomes of RFI

To optimize the fluorimetric response, a series of trials was performed to identify the ideal volume of Acid Red 87 reagent required to maximize the ΔFI signal. As illustrated in Fig. 4, the most effective enhancement of ΔFI was achieved with 1.2 mL of Acid Red 87 solution. Insufficient reagent volumes yielded weak signals, indicating that the complexation reaction was incomplete. In contrast, excessive amounts of Acid Red 87 appeared to diminish the signal, likely due to self-aggregation phenomena that interfere with its reactivity.

### Dispersing solvent

To evaluate the most suitable dispersing medium for the fluorimetric system, a range of solvents—including acetone, various alcohols, and distilled water—was tested. Among these, distilled water produced the greatest enhancement in fluorescence intensity (ΔFI), as shown in Fig. 4. Its selection was further supported by its environmentally benign nature, making it a preferable alternative to organic solvents. The instability observed in the complex when exposed to organic solvents highlighted the importance of solvent choice. Distilled water’s superior performance was attributed to its high polarity index (10.2) and dielectric constant (80.2), which facilitated efficient dilution and maintained system integrity. Unlike organic solvents, which vary in dielectric properties and may hinder complex formation, distilled water ensured complete miscibility and compatibility with the aqueous environment of the system.

### Time impact on the system stability

The DLZ- Acid Red 87 complex exhibited immediate formation upon reagent admixture, achieving kinetic equilibrium within the mixing time (< 30 s). This complex demonstrated exceptional temporal stability, maintaining invariant fluorescence quenching intensity for > 60 min under ambient conditions. This strong performance makes it dependable for use in fast pharmaceutical quality checks and biological monitoring, where samples need to be processed over longer periods.

### Validation

Analytical validation constitutes a critical procedure for establishing the dependability and fitness-for-purpose of a novel analytical methodology. This process necessitates a structured and thorough assessment of key performance attributes, such as accuracy, precision, linearity, limits of detection, and quantitation. Through the application of statistical analysis and stringent experimental testing, validation furnishes documented evidence regarding the method’s robustness and its capacity to generate consistent, reliable, and reproducible results. Furthermore, the validation exercise aids in pinpointing potential sources of error or analytical interference, thereby facilitating the implementation of necessary methodological refinements and precautions to ensure optimal performance. Consequently, analytical validation serves a foundational role in substantiating the credibility of the new method and broadening its applicability across diverse analytical contexts.

### System linearity and sensitivity

The assessment of linearity and range is paramount in determining an analytical method’s performance for generating reliable results across varying concentrations within a defined interval. Linearity characterizes the method’s capacity to demonstrate a proportional relationship between the analyte concentration (DLZ) and the instrumental response (ΔFI). Validation of linearity confirms the method’s suitability for accurate quantification over the intended concentration span. This is evaluated by analyzing standard solutions of known DLZ concentrations, plotting the response (ΔFI) against concentration, and statistically analyzing the resulting data. Key regression parameters, including the correlation coefficient, slope, and intercept (detailed in Table 1), provide critical insight into the method’s accuracy for DLZ measurement. Furthermore, the method’s range delineates the interval between the upper and lower concentration limits that can be quantified with reliability. Establishing this range is essential for defining the method’s applicability and identifying constraints on its accuracy within specific concentration boundaries.

**Table 1:** Regression parameters provided for the created system

Parameters	Values
Linear range (ng/mL)	50–1100
Slope	4.42
Intercept	1148.5
SD of intercept (S_a_)	20.8
Correlation coefficient (r)	0.9995
Determination coefficients (r^2^)	0.9991
Number of determinations	6
Limit of quantitation (ng/mL)	47
Limit of detection (ng/mL)	15.5

The limits of detection (LOD) and quantitation (LOQ) were calculated using established formulae: LOD = (3.3 × σ)/S and LOQ = (10 × σ)/S, where σ represents the standard deviation of the y-intercept residuals and S is the slope of the calibration curve. Based on this calculation, the LOD was determined to be 15.5 ng mL⁻¹, and the LOQ was estimated to be 47 ng mL⁻¹.

### Accuracy and precision

Accuracy represents a fundamental validation parameter under ICH (International Council for Harmonisation) guidelines, confirming an analytical method’s capability to generate results approximating the true value. Accuracy validation necessitates comparing method-derived results against a reference standard or known value. The method is deemed accurate if results reside within predefined acceptable limits of this reference. ICH guidelines outline multiple strategies for accuracy assessment, including recovery experiments, standard addition techniques, and comparison with certified reference materials. These approaches substantiate the method’s reliability in quantifying the analyte within pharmaceutical formulations. Within the present investigation, accuracy was verified via a recovery study applying the general assay procedure to four distinct DLZ concentrations. As detailed in Table 2, the system exhibited excellent accuracy, demonstrated by acceptable recovery percentages, standard deviations, and percentage errors.

**Table 2:** Accuracy of the created system at four DLZ concentrations

Conc. level (ng/mL)	Recovery^a^ % ± SD	% Error
200	101.14 ± 1.49	1.14
600	98.06 ± 1.28	1.94
800	98.67 ± 0.87	1.33
1000	101.15 ± 0.53	1.15

^a^Mean of three DLZ determinations and SD is the standard deviation

Precision characterizes the consistency and reproducibility of measurements obtained using an analytical method. Evaluating precision is essential for researchers and regulators to ascertain the repeatability of results derived from multiple replicate analyses. This assessment is critical for ensuring the dependability of analytical data throughout method development and evaluation. ICH guidelines specify statistical methodologies and acceptance criteria for precision determination, notably the calculation of the relative standard deviation (RSD). In this study, method precision was assessed at three different DLZ concentrations, with RSD calculated for each recovery percentage. The results presented in Table 3 confirm the recommended fluorimetric method achieved RSD values below 2% across precision levels, indicating high precision.

**Table 3:** Precision of the created system at two levels

Conc. level (ng/mL)	Intraday precision		Interday precision	
	Recovery^a^ % ± SD	RSD %	Recovery^a^ % ± SD	RSD %
300	98.51 ± 1.07	1.09	101.95 ± 1.52	1.49
500	100.54 ± 1.20	1.19	101.69 ± 0.74	0.73
900	100.10 ± 0.97	0.96	99.03 ± 1.30	1.31

^a^Mean of three DLZ determinations and RSD is the relative standard deviation

### Robustness

Robustness describes an analytical method’s capacity to maintain consistent performance when subjected to deliberate, minor alterations in critical procedural parameters. This assessment evaluates the method’s dependability and precision under conditions incorporating intentional, controlled variations. Investigating robustness during method development is essential. By systematically modifying factors such as pH, dye volume, and buffer volume, critical variables potentially influencing method performance can be identified. Robustness testing thus assesses the method’s tolerance to typical fluctuations encountered in routine laboratory environments or slight deviations in experimental protocol. The data compiled in Table 4 indicate significant resilience, as no substantial deviations in performance were detected across the tested variations.

**Table 4:** Evaluating the robustness of the proposed DLZ assay method

Parameter	Value	Recovery^a^ % ± SD	RSD %
pH	3.7	98.08 ± 1.73	1.76
	3.9	99.16 ± 1.55	1.56
Buffer volume (mL)	1.2	101.66 ± 1.81	1.78
	1.4	100.08 ± 1.51	1.51
Dye volume (mL)	1.1	98.43 ± 1.88	1.91
	1.3	100.93 ± 1.15	1.14

^a^Mean of three replicate DLZ measurements, SD = Standard deviation

### Reaction stoichiometry

**Fig. 5:**
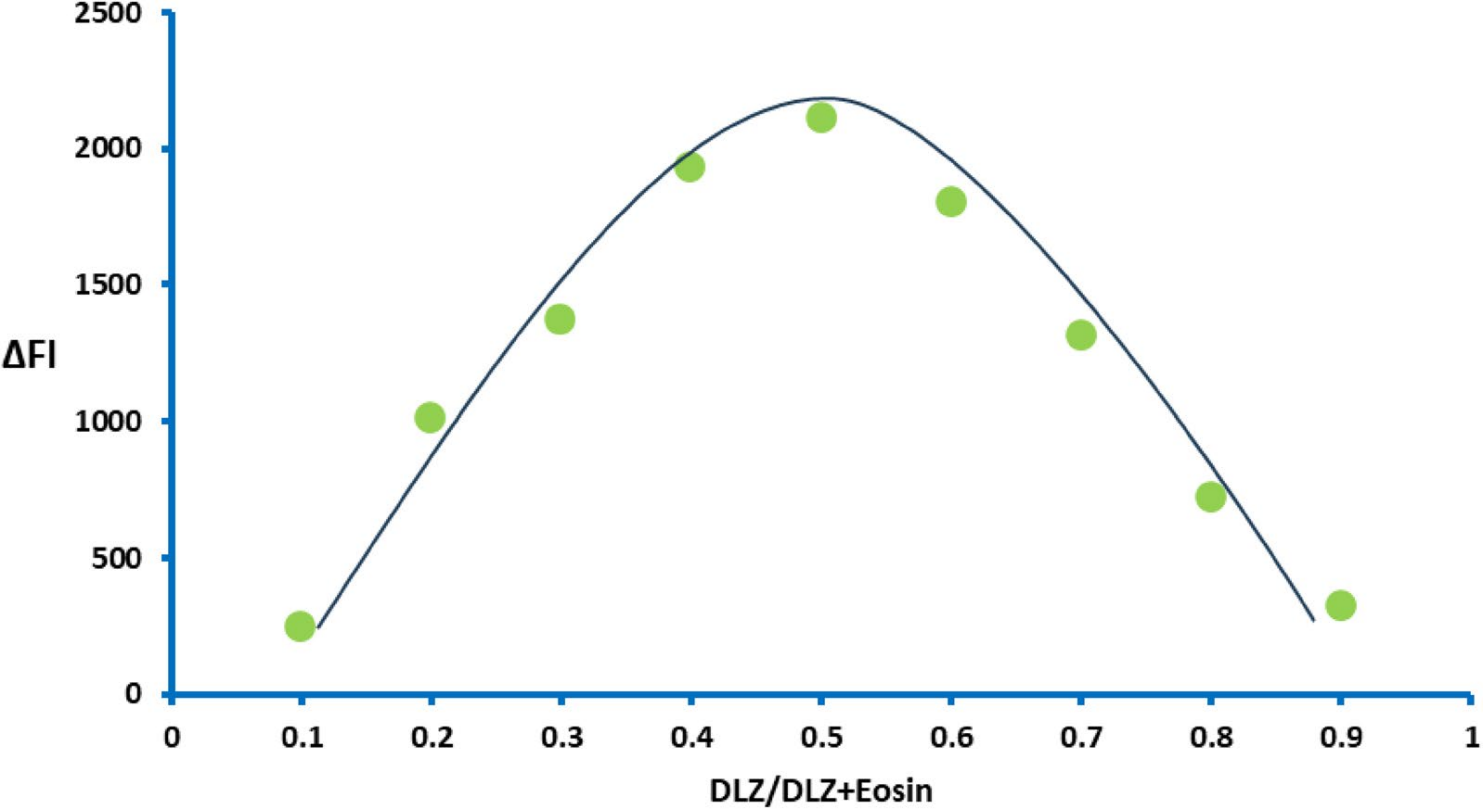
Job‘s plot of continuous variation for the complex formation between Acid Red 87 and DLZ

The composition of the complex between Acid Red 87 and DLZ was investigated using the continuous variation method (Job’s plots). Equimolar solutions of both the dye and the drug were prepared and incorporated into the general procedure using complementary volumes of both solutions. The ΔFI was measured for each solution, and the decrease in fluorescence at 545.8 nm was plotted against the drug mole fraction. A maximum value was obtained in the plot at a drug mole fraction of about 0.5 (Fig. 5). This value confirmed the formation of a binary complex that contains drug: dye in a ratio of 1:1.

### Application

Applying validated analytical methods to pharmaceutical dosage forms is essential for acquiring critical data on product quality, composition, and performance. In the present investigation, the developed fluorimetric assay was successfully employed to analyze commercial Altiazem 60 mg tablets and Diltiazem 90 mg capsules. These DLZ-containing formulations were subjected to the established assay protocol. Statistical comparisons between percent recovery results obtained via the new fluorimetric system and a reference method [25] were performed using t-tests and F-tests. At the 95% confidence level, the calculated t- and F-values did not exceed the critical values, demonstrating no statistically significant differences in precision or accuracy between the two methods (Table 5). The current fluorimetric approach presents practical advantages, including operational simplicity, high sensitivity, cost-effectiveness, and readily available reagents, establishing it as a viable alternative for routine DLZ quantification in pharmaceutical products. Moreover, no need for derivatization heating, faster analysis time, and the exclusive use of water instead of organic solvents.

**Table 5:** Dosage form analysis of DLZ and comparison with the reported method

Dosage form	Current method	Reported method [24]		
	Recovery^a^ ± SD	Recovery^a^ ± SD	t- test value^b^	F-value^b^
Altiazem 60 mg tablets	99.41 ± 1.04	99.26 ± 0.93	0.25	1.24
Diltiazem 90 mg capsules	98.93 ± 1.79	98.62 ± 1.13	0.33	2.50

^a^The value is the average of five determinations for both the proposed and reported methods

^b^Tabulated values at 95% confidence limit are t = 2.306, F = 6.338

### Method greenness

**Fig. 6:**
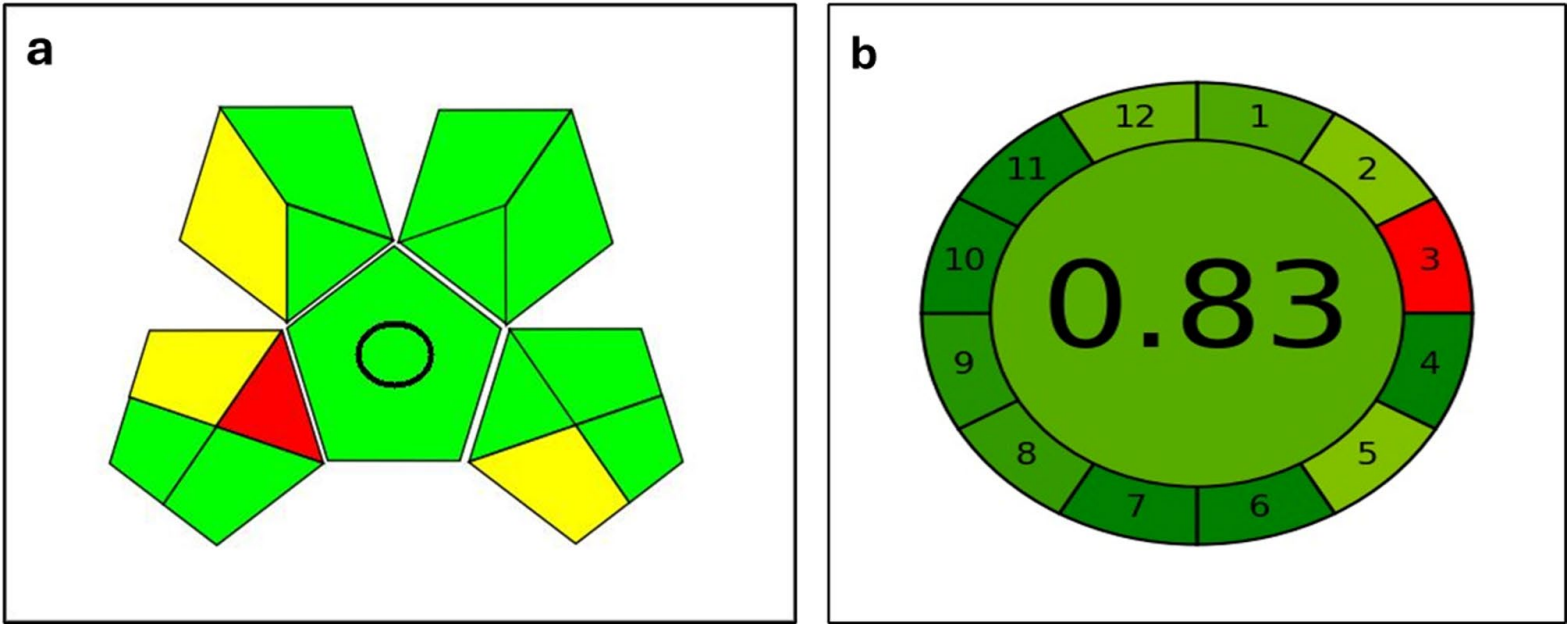
Greenness evaluation of the current method using GAPI index (**a**) and AGREE (**b**) tools

The imperative of green chemistry within analytical methodologies is underscored by its focus on designing processes and products that mitigate environmental harm and safeguard human health, as defined by the Environmental Protection Agency. This is operationalized by adopting non-toxic, renewable solvents and developing efficient, low-impact instrumental techniques [26, 27]. Consequently, standardized assessment tools such as AGREE and GAPI have been developed to quantitatively evaluate a method’s ecological footprint; AGREE employs a clock-shaped pictogram where a score closer to 1 indicates superior greenness, while GAPI uses a color-coded pentagram system [28–30]. The described spectrofluorometric method for analysis exemplifies these principles, achieving a high greenness rating due to its elimination of organic solvents, use of low-energy equipment, high throughput, and simple preparation without derivatization, as visually confirmed in its AGREE and GAPI assessments (Fig. 6).

## Conclusion

This research details the development of an innovative spectrofluorimetric method for the quantification of diltiazem (DLZ). The approach relies on the formation of an ion-association complex to measure DLZ within an acidic aqueous medium, demonstrating a linear working range of 50 to 1100 ng/mL. Acid Red 87 was selected as the complexing reagent due to its distinct advantages. A primary benefit is its enhanced environmental compatibility; its ready water solubility facilitates straightforward detection in aqueous systems. The complex formation between DLZ and the deprotonated hydroxyl group of Acid Red 87 occurs directly in water, eliminating extraction steps and consequently reducing analysis time and operational costs. This renders the technique both straightforward and expeditious. Furthermore, employing water as the sole solvent minimizes environmental impact by circumventing the use of volatile organic compounds. The method proved effective for quantifying DLZ in diverse matrices, including pharmaceutical tablets and capsules demonstrating high specificity with minimal matrix interference. Consequently, this procedure exhibits significant potential for adoption in research laboratories, pharmaceutical quality control, and clinical settings for therapeutic drug monitoring.

## Data Availability

The data will be available upon reasonable request from the corresponding author.
